# New advances in the diagnosis and treatment of autism spectrum disorders

**DOI:** 10.1186/s40001-024-01916-2

**Published:** 2024-06-10

**Authors:** Lei Qin, Haijiao Wang, Wenjing Ning, Mengmeng Cui, Qian Wang

**Affiliations:** 1 Department of Rehabilitation, The Second Affiliated Hospital of Shandong First Medical University, Taian, Shandong China; 2 Department of Intensive Care Medicine, Feicheng People’s Hospital, Taian, Shandong China; 3 Department of Central Laboratory, The Affiliated Taian City Central Hospital of Qingdao University, Taian, China

**Keywords:** Autism spectrum disorder (ASD), Diagnostic methods, Treatment strategies, Precision medicine, Emerging biotechnology

## Abstract

Autism spectrum disorders (ASD) are a group of neurodevelopmental disorders that affect individuals' social interactions, communication skills, and behavioral patterns, with significant individual differences and complex etiology. This article reviews the definition and characteristics of ASD, epidemiological profile, early research and diagnostic history, etiological studies, advances in diagnostic methods, therapeutic approaches and intervention strategies, social and educational integration, and future research directions. The highly heritable nature of ASD, the role of environmental factors, genetic–environmental interactions, and the need for individualized, integrated, and technology-driven treatment strategies are emphasized. Also discussed is the interaction of social policy with ASD research and the outlook for future research and treatment, including the promise of precision medicine and emerging biotechnology applications. The paper points out that despite the remarkable progress that has been made, there are still many challenges to the comprehensive understanding and effective treatment of ASD, and interdisciplinary and cross-cultural research and global collaboration are needed to further deepen the understanding of ASD and improve the quality of life of patients.

Autism spectrum disorders (ASD) are a broad group of neurodevelopmental disorders that affect an individual's social interactions, communication skills, and behavioral patterns [1, 2]. The characteristics of ASD vary significantly between individuals, from mild social impairments to severe communication and behavioral problems, a diversity that reflects the use of the term “spectrum” [3]. Although the exact causes of ASD are not fully understood, research suggests that both genetic and environmental factors play a key role in its development [4].

## Characteristics of ASD

### Difficulties in social interaction

Individuals with ASD often exhibit significant difficulties in social interactions. These difficulties may include difficulty understanding the feelings and intentions of others, maintaining eye contact and facial expressions, and adapting to social norms and expectations. Individuals with ASD may experience challenges in establishing and maintaining friendships, they may not understand the two-way nature of social interactions, or they may feel uncomfortable sharing interests and activities [5].

### Communication disorders

Communication deficits are another core feature of ASD. This may manifest itself in delays in language development, including delays in uttering first words or simple sentences. Some individuals with ASD may not use language to communicate at all. Even among individuals with ASD who have normal language skills, they may have difficulty using language in conversations to communicate thoughts, feelings, or needs. In addition, nonverbal communication, such as the understanding and use of body language and facial expressions, may also be affected [6].

### Repetitive behaviors and interests

Individuals with ASD often display restricted, repetitive patterns of behavior and interests. These may include a strong fixation on specific topics or activities, repetitive body movements (e.g., rocking, clapping), and an overreliance on daily routines. These repetitive behaviors are sometimes seen as a way of self-soothing or as an attempt to control an environment that otherwise feels unpredictable and overwhelming to them [7].

### Sensory sensitivity

Many individuals with ASD have abnormalities in sensory processing and may have very strong or delayed responses to sound, light, touch, taste or odor. For example, some individuals with ASD may find background noises in their everyday environment unusually harsh, or they may not notice pain or other bodily sensations [8].

## Epidemiologic profile of ASD

According to the World Health Organization (WHO), the average prevalence of ASD among children globally is approximately 1% [9]. However, this figure varies significantly between regions and countries. For example, the Centers for Disease Control and Prevention (CDC) reports that the prevalence of ASD among 8-year-olds in the U.S. is 1 to 54. ASD is significantly more prevalent in males than females, at a ratio of approximately 4:1 [10]. This gender difference may reflect differences in genetic susceptibility and/or gender bias in the diagnostic process. Early diagnosis is key to improving developmental outcomes for children with ASD. Despite this, many children are not diagnosed by age 3. The CDC reports that most children are first evaluated for ASD by age 4, but diagnosis may occur later. Research suggests that ASD is highly heritable, but multiple genetic variants are associated with disease risk and environmental factors also play a role [11]. For example, there is an increased risk of ASD in preterm and low birth weight infants. Socioeconomic factors influence ASD diagnosis and treatment access. Families of lower socioeconomic status may face greater challenges, including barriers to accessing early intervention services, etc. ASD is a global public health problem, and its incidence, time to diagnosis, and treatment access are influenced by multiple factors [12]. Ongoing epidemiologic research and the advancement of a deeper understanding of ASD are critical to the development of effective prevention, diagnosis, and interventions.

## Historical background

### Early history of research and diagnosis of ASD

The concept of ASD was first clearly defined in the 1940s, when a group of children exhibiting extreme self-isolation and lack of responsiveness to the environment was first described by American psychiatrist Leo Kanner [13]. Almost simultaneously, Austrian child psychologist Hans Asperger described a similar but higher level of functioning in a condition that came to be known as Asperger’s syndrome [14]. These two independent studies laid the foundation for the modern understanding of ASD. For the first few decades, ASD was considered extremely rare and was often confused with schizophrenia. Due to a lack of in-depth understanding of ASD, early diagnostic criteria were unclear and treatment was largely limited to behavioral interventions and psychotherapy. Over time, researchers began to pay more attention to the genetic and neurobiological underpinnings of ASD, thus contributing to a more comprehensive understanding of this complex condition. Since the 1990s, the diagnosis of ASD has risen significantly, as diagnostic criteria have continued to be refined and public awareness has increased. This period has also witnessed an increased awareness of the importance of early diagnosis and intervention for ASD, which has led to significant improvements in the prognosis and quality of life for many children and adults with ASD [15].

### Evolution of research paradigms

The research paradigm for ASD has undergone a remarkable evolution since the mid-twentieth century, a process that reflects a deepening of the understanding of ASD as well as advances in scientific research methods [16]. In the early stages, ASD research focused on behavioral observations and psychoanalysis, when ASD was often mistaken for an emotional disorder due to an indifferent mother. During this period, understanding of ASD was relatively limited and treatments focused primarily on psychotherapy and behavior modification. Into the second half of the twentieth century, with advances in genetics and neuroscience, researchers began to explore the biological basis of ASD. This marked a shift from a psychosocial to a biomedical model, and the focus of research gradually shifted to genetic factors and abnormalities in brain structure and function. Through a large number of family and twin studies, scientists found that ASD has a high genetic predisposition, while neuroimaging studies revealed the specificity of brain development in ASD patients. In the twenty-first century, with the application of bioinformatics and high-throughput gene sequencing technology, the study of ASD has entered a new stage [17]. Researchers have not only been able to identify specific genetic variants associated with ASD, but have also begun to explore the interaction between environmental factors and genetic susceptibility. In addition, the adoption of interdisciplinary research approaches, such as combining neuroscience, genetics, psychology, and computational modeling, has provided new perspectives for understanding the complexity of ASD.

Recently, the concepts of precision medicine and personalized treatment strategies have been introduced to the study of ASD, aiming to develop customized intervention programs based on each patient’s genetic background and symptom profile. With advances in technology and improved methods of data analysis, future research on ASD is expected to reveal more knowledge about its pathomechanisms and provide more effective support and treatment for patients with ASD.

## Etiologic studies

### Genetic factors

#### Monogenic genetic cases

The etiology of ASD is multifactorial, involving a complex interaction of genetic and environmental factors. Although most cases of ASD are thought to be the result of polygenic interactions, there are some cases that are directly associated with variations in a single gene, and these are referred to as monogenic genetic cases. Monogenic genetic cases provide an important window into understanding the genetic basis of ASD, although they represent a relatively small proportion of all ASD cases [18]. A number of specific genetic syndromes, such as fragile X syndrome, tuberous sclerosis, 15q11-q13 duplication syndrome, and Rett syndrome, have been found to be associated with a higher risk of ASD. These conditions, often caused by mutations or abnormalities in a single gene, can lead to significant differences in brain development and function, thereby increasing the probability of an ASD phenotype. Fragile X syndrome is one of the most common forms of inherited intellectual disability and the single-gene disorder known to be most strongly associated with ASD. It is caused by a repeat expansion on the FMR1 gene [19]. Tuberous sclerosis (TSC) is an inherited disorder that affects multiple systems and is caused by mutations in the TSC1 or TSC2 genes, and the prevalence of ASD is higher in patients with TSC. 15q11-q13 duplication syndrome (Dupuy 15q syndrome) involves a region of chromosome 15, the duplication of which is associated with an increased risk of ASD [20]. Rett syndrome, which predominantly affects females, is caused by mutations in the MECP2 gene, and patients often exhibit some of the features of ASD, such as impaired social interactions [21]. The association of these classical candidate genes with ASD is summarized in Table 1.

**Table 1:** Classic candidate genes associated with ASD

Gene	Expression trend	Involved signaling pathways	Biological functions
SHANK3 [22]	Downregulated	mTOR pathway, synaptic plasticity	Synapse formation and function
NRXN1 [23]	Downregulated	Reelin pathway, synapse formation	Neuronal adhesion, synapse formation |
NLGN3[24]	Up- or downregulated	mTOR pathway, synaptic plasticity	Synapse formation and function
MECP2 [25]	Up- or downregulated	mTOR pathway, transcriptional regulation	Gene expression regulation, neuronal development
FMR1 [26]	Downregulated	mGluR5 pathway, mRNA transport	Synaptic plasticity, cognitive development
PTEN [27]	Downregulated	PI3K/AKT/mTOR pathway	Cell proliferation, synaptic development
CHD8[28]	Downregulated	Wnt/β-catenin pathway	Chromatin remodeling, Wnt signaling
FOXP1 [29]	Downregulated	–	Language and cognitive development

The discovery of these monogenic genetic cases is not only crucial for understanding the genetic mechanisms of ASD, but also potentially valuable for the development of interventional and therapeutic strategies targeting specific genetic variants. However, even in these cases, the expression of the genetic variants showed a degree of heterogeneity, suggesting that the diversity of phenotypic features and clinical manifestations, even in monogenic genetic cases, may be influenced by other genetic and environmental factors. Therefore, an in-depth study of these conditions will not only improve our understanding of the genetic basis of ASD, but also provide clues for the development of more personalized therapeutic strategies.

#### Multigene interactions

The development of ASD is widely recognized as a result of the interaction of genetic and environmental factors, with polygenic interactions occupying a central position in the genetic background of the disease. Unlike monogenic cases, polygenic interactions involve variants or polymorphisms in multiple genes that together increase the risk of ASD. These genetic variants may contribute a smaller effect in each individual, but when acting together they can significantly increase the probability of ASD development [30]. Current research suggests that no single gene can explain all cases of ASD. Instead, hundreds of genetic loci have been identified that are associated with an increased risk of ASD. These genes are often involved in key processes such as brain development, neuronal signaling, and intercellular communication, suggesting that ASD involves extensive regulation of brain function and structure. The complexity of multigene interactions means that genetic studies of ASD require large-scale genomic data and sophisticated statistical methods to reveal those genomic variants that increase risk.

Meta-analyses of large-sample genome-wide association studies (GWAS) have identified several consistently replicated ASD risk gene loci, such as those in the chromosomal regions 3p21, 5p14, 7q35, and 20p12. These loci contain genes like CNTN4, CNTNAP2, and NRXN1, which play crucial roles in neurodevelopment and synaptic function, particularly in processes such as synaptic adhesion and neurotransmission. These findings provide a more robust understanding of the genetic architecture of ASD and highlight the importance of integrating genetic findings with functional studies to advance our understanding of the disorder. They also have implications for future research, such as the development of personalized diagnostic and therapeutic strategies based on an individual's genetic profile. Through genome-wide association studies (GWAS) and other genomic approaches, scientists are gradually unraveling the genetic landscape of this complex disease. Understanding the impact of multiple gene interactions on ASD not only helps us understand its genetic basis, but also opens up the possibility of developing personalized treatment and intervention strategies [31].

### Environmental factors

#### Maternal exposure

Exposure during pregnancy refers to a mother’s exposure to specific environmental factors or substances during fetal development, which may increase the child's risk of developing ASD in the future. These exposures include certain prescription medications (e.g., anti-seizure medications and opioids), environmental pollutants (e.g., heavy metals and air pollutants), infections (e.g., rubella and influenza viruses), and poor nutrition or deficiencies in specific nutrients (e.g., folic acid). These factors may increase the risk of ASD by affecting fetal brain development and the maturation process of the nervous system. Understanding the effects of exposure during pregnancy can help to take preventive measures to reduce the incidence of ASDs [32].

#### Effects of early developmental stages

The early developmental stages of ASD are influenced by a variety of factors that include genetic predisposition, environmental exposures, and early life experiences. During a child's early development, the brain experiences rapid growth and the formation of neural networks. Any disruption during this critical period may interfere with the proper development of brain structure and function, thereby increasing the risk of ASD. For example, very early lack of social interaction, delayed language development or abnormal sensory processing may be early signs of ASD. These developmental abnormalities reflect difficulties in the brain’s nervous system in processing information, making connections and adapting to environmental changes. Early identification and intervention are essential to promote optimal development in children with ASD [33].

#### Genetic–environmental interactions

**Fig. 1:**
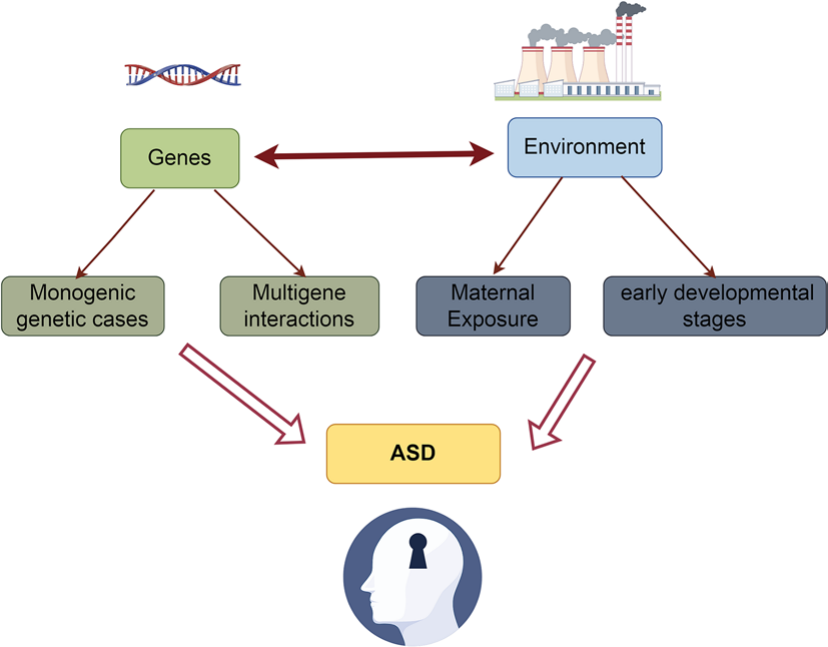
Genetic–environmental interactions

The genetic–environmental interactions are summarized in Fig. 1. ASD develops as a result of the interaction between genetic and environmental factors, and this interaction reflects the complexity of the combination of genetic background and external environmental factors that influence ASD risk. Specifically, certain genetic susceptibilities may be activated in response to environmental triggers, leading to the development of ASD. For example, genetic variants may make individuals more sensitive to certain environmental exposures (e.g., substance use during pregnancy, environmental pollutants, or maternal nutritional status), which together may increase the risk of ASD by acting on key brain developmental stages [34]. This complex genetic–environmental interaction underscores the need to understand multifactorial etiological models of ASD and the importance of developing personalized intervention strategies.

## Advances in diagnostic methods

### Traditional diagnostic methods

Traditional diagnostic methods for ASD rely heavily on detailed assessments of behavior and developmental history. These assessments are usually conducted by specialized health care providers such as pediatricians, neuropsychologists, or psychiatrists. The diagnostic process encompasses direct observation of the child as well as in-depth interviews with parents or caregivers to gather information about the child's social interactions, communication skills, and behavioral patterns [35]. Diagnostic tools include, but are not limited to, the Childhood Autism Rating Scale (CARS), the Autism Diagnostic Observation Scale (ADOS), and the Autism Diagnostic Interview-Revised (ADI-R). These tools are designed to identify core symptoms of ASD, such as social communication deficits and repetitive behaviors or interests. In addition, the doctor may perform a series of developmental or cognitive assessments to rule out other conditions that may explain the child’s behavior, such as language disorders or other neurodevelopmental disorders [36]. While these traditional diagnostic methods are highly effective in recognizing ASD, they rely on subjective assessments and the experience of the professional, and therefore may have some degree of variability. In recent years, with a deeper understanding of ASDs, new diagnostic techniques and methods are being developed and adopted to improve diagnostic accuracy and efficiency.

### Latest diagnostic techniques and tools

#### Genetic testing

Genetic testing for ASD is a method of identifying risks associated with ASD by analyzing genetic variants in an individual's DNA. This testing looks for specific genetic variants that have been linked by scientific research to the development of ASD. Although the genetic background of ASD is extremely complex, involving multiple genes and the interaction of genes with environmental factors, variants in specific genes have been identified as having a significant impact on ASD risk [37]. For example, variants in the SHANK3 gene are associated with Phelan–McDermid syndrome, and patients with this syndrome often exhibit ASD features. Variants in the FMR1 gene are responsible for fragile X syndrome, which is the most common single-gene cause of ASD known to be associated with ASD. Mutations in the MECP2 gene have been associated with Rett syndrome, and patients with Rett syndrome often exhibit ASD condition. In addition, variants in the NRXN1 and NLGN3/4 genes have been found to increase the risk of ASD [38]. Genetic testing can help provide more precise diagnostic information, and in those cases of ASD where the cause is unknown, it may even reveal the underlying genetic cause. This will not only help to understand the genetic mechanisms of ASD, but also provide more targeted intervention and support strategies for patients and families.

#### Neuroimaging

Neuroimaging techniques in the study of ASD provide a non-invasive way to explore changes in brain structure and function, helping scientists better understand the biological basis of ASD. These techniques include functional magnetic resonance imaging (fMRI), structural magnetic resonance imaging (sMRI), diffusion tensor imaging (DTI), and positron emission tomography (PET). Through these neuroimaging techniques, researchers are able to observe structural and functional differences in specific regions and networks of the brain in individuals with ASD [39]. For example, fMRI can reveal patterns of brain activity when performing specific tasks, helping to understand the impairments in social, language, and cognitive functioning in individuals with ASD. dTI focuses on the microstructure of the brain’s white matter, revealing the connections of bundles of nerve fibers, which can help to study neural connectivity issues in ASD. PET scans, on the other hand, are able to assess the activity of specific chemicals in the brain, providing clues to study the neurochemical basis of ASD [40]. With these advanced neuroimaging techniques, researchers will not only be able to delve deeper into the neurodevelopmental abnormalities of ASD, but also identify possible novel therapeutic targets that can provide a scientific basis for developing more effective interventions. However, while these techniques provide valuable perspectives in understanding ASD, a complete understanding of the complexity of the brain remains a challenge for future research.

#### Early screening methods

Recently, the field of early screening for ASD has witnessed the application of a number of innovative techniques designed to improve the accuracy and convenience of screening. One notable new approach is the use of artificial intelligence (AI) and machine learning techniques to analyze children's behavioral videos and biomarkers. By training algorithms to recognize specific behavioral patterns and physiological signals associated with ASD, these technologies can help physicians and researchers identify potential ASD symptoms earlier [41]. Another area of innovation is eye-tracking technology, which assesses children’s social and cognitive development by analyzing their eye movement patterns when viewing pictures or videos. Studies have shown that the eye movement patterns of children with ASD while viewing social scenes differ from those of typically developing children, providing a non-invasive window for early screening [42]. The application of these state-of-the-art technologies not only improves the efficiency and accessibility of early screening, but also provides new perspectives for understanding the complexity and individual differences in ASD [43]. Although these approaches are still in the research and development stage, they demonstrate the great potential of utilizing technological advances to improve the process of ASD screening and diagnosis. With further validation and refinement of these techniques, it is expected that they will make a significant contribution to the early identification and intervention of ASD in the future.

## Treatment approaches and intervention strategies

### Behavioral and educational interventions

#### Applied behavior analysis (ABA)

Applied behavior analysis (ABA) is an intervention approach based on the principles of behavioral psychology that is widely used in the treatment of children with autism spectrum disorders (ASD). ABA works to understand and improve specific behaviors, particularly to enhance social, communication, academic skills, and daily living skills, while reducing maladaptive behaviors. It helps individuals learn new skills and behaviors by systematically applying reinforcement strategies that encourage and reward desired behaviors [44]. ABA therapy is highly individualized and customized to each child’s specific needs and abilities. Treatment planning begins with a detailed behavioral assessment to identify target behaviors and intervention strategies. Learned behaviors are then reinforced and cemented through one-on-one teaching sessions using positive reinforcement. ABA also emphasizes the importance of data, which is collected and analyzed on an ongoing basis by the therapist to monitor progress and adjust the treatment plan as necessary [45]. Research has shown that ABA is an effective way to improve social interactions, communication skills, and learning in children with ASD. Through early and consistent intervention, ABA can significantly improve the independence and overall quality of life of children with ASD. Although ABA treatment requires a commitment of time and resources, the long-term benefits it brings to children with ASD and their families are immeasurable.

#### Social skills training

Social skills training (SST) for children with autism spectrum disorders (ASD) is an intervention designed to improve their ability to interact socially in everyday life. This training focuses on teaching children with ASD the ability to understand social cues, establish effective communication skills, and develop friendships. Through SST, children learn how to recognize and interpret other people's facial expressions, body language, and social etiquette, which are essential for building positive relationships [46]. Social skills training typically includes a series of structured instructional activities such as role-playing, social stories, interactive group exercises, and peer modeling. These activities are designed to provide practice in real-world social situations in a supportive and interactive manner, helping children with ASD learn and practice new skills in a safe environment [47]. In addition, SST can include teaching emotion management and conflict resolution skills to help children with ASD better understand and express their emotions and cope with challenges in social interactions. Through regular and consistent practice, children with ASD can improve their self-confidence, increase their social engagement, and ultimately improve their social competence and quality of life. SST has been shown to be significantly effective in enhancing social adjustment and interpersonal interactions in children with ASD [48].

### Medical treatment

#### Medication

While there is no cure for ASD, certain medications can be used to manage specific symptoms associated with ASD, such as behavioral problems, attention deficits, anxiety, and mood swings that are common in individuals with autism. Medication is often used as part of a comprehensive intervention program designed to improve the quality of life and daily functioning of the patient [49]. Medications commonly used for ASD symptom management include antipsychotics, antidepressants, stimulants, and anxiolytics. For example, two antipsychotics, risperidone and aripiprazole, have been approved by the FDA for the treatment of stereotypic and aggressive behavior in children and adolescents with ASD. In addition, selective serotonin reuptake inhibitors (SSRIs) may be helpful in managing anxiety and depressive symptoms in individuals with ASD.

Importantly, medication needs to be closely monitored by a physician to ensure the effectiveness and safety of the medications, as they may have side effects. We have summarized the research evidence on the efficacy and safety of commonly used medications in ASD, including antipsychotics for treating irritability and aggression, antidepressants for co-occurring anxiety and depression, and other medications such as stimulants and melatonin. While these medications can be helpful in managing specific symptoms, they also carry potential side effects and risks, such as weight gain, metabolic disturbances, and behavioral activation. Therefore, a thorough diagnostic evaluation, individualized treatment planning, close monitoring, and regular follow-up are essential when considering pharmacotherapy for individuals with ASD. The decision to medicate should be based on an individualized assessment that takes into account the patient’s specific needs, the severity of symptoms, and possible side effects. At the same time, pharmacological treatments are often used in combination with non-pharmacological treatments such as behavioral interventions and educational support to achieve optimal therapeutic outcomes [50].

#### Biofeedback and neuromodulation

Biofeedback and neuromodulation are innovative approaches that have been explored in recent years in the treatment of ASD, aiming to reduce ASD symptoms by improving brain function. Biofeedback techniques enable individuals to learn how to control physiological processes that are not normally under conscious control, such as heart rate, muscle tension, and brainwave activity. Through real-time feedback, patients can learn how to regulate their physiology, resulting in improved concentration, reduced anxiety, and improved emotional regulation. Neuromodulation, specifically transcranial magnetic stimulation (TMS) and transcranial direct current stimulation (tDCS), affects neural activity in the brain through external stimulation. tMS utilizes a magnetic field to affect neuronal activity in specific areas of the brain, while tDCS modulates neuronal excitability by applying a weak electrical current. These methods have been studied for improving social communication skills and reducing stereotypical behaviors in people with ASD [51].

Biofeedback helps individuals develop self-regulation skills by providing real-time feedback on physiological states, while neuromodulation techniques like TMS and tDCS modulate cortical excitability and neural plasticity in aberrant circuits implicated in ASD. Current research suggests potential benefits of these techniques in improving emotional regulation, social functioning, and cognitive performance, but mixed results highlight the need for larger, well-controlled trials to validate efficacy, safety, and optimal protocols. Despite challenges, these techniques show promise as adjunctive therapies in the comprehensive management of ASD, warranting further research to guide their translation into clinical practice. Although biofeedback and neuromodulation show potential in the treatment of ASD, research on these techniques is currently in its infancy. More clinical trials and studies are needed to evaluate their effectiveness, safety, and long-term effects and to determine which patients may benefit from these interventions. Nevertheless, as non-pharmacologic treatments, they offer promising complementary options to the comprehensive treatment of ASD.

### Emerging intervention approaches

#### Technology-assisted interventions

Technology-assisted interventions have become an important development in the field of ASD treatment in recent years, providing new ways for children with ASD to learn and communicate. These interventions utilize computers, tablets, smartphone apps, and virtual reality technology to design a range of interactive learning tools and games designed to improve social skills, communication, and cognitive functioning in children with ASD [52]. A key advantage of technology-assisted interventions is their ability to provide highly personalized learning experiences. Software and applications can be adapted to a child's specific needs and interests, ensuring that learning content is both engaging and appropriate to the individual's developmental level. In addition, the feedback provided by technology is often immediate and consistent, helping children with ASD to better understand and process information. The use of virtual reality technology, by simulating social situations, provides a safe and controlled environment for children with ASD to practice social interaction and problem-solving skills, which is often difficult to achieve in traditional educational and therapeutic settings [53]. Although technology-assisted interventions have demonstrated great potential, research on their long-term effects and optimal implementation is still ongoing. To maximize the benefits of these tools, it is often recommended that technology-assisted interventions be used in conjunction with other therapeutic approaches to provide a comprehensive intervention program.

#### Diet and nutrition interventions

Dietary and nutritional interventions have received increasing attention in the treatment of ASD, based on the observed potential link between nutritional imbalances and ASD symptoms. This intervention approach aims to improve the behavioral performance and overall health of children with ASD by optimizing their diet. Specific strategies include restricting certain foods that may exacerbate symptoms, such as gluten and lactose, as well as increasing intake of foods rich in essential nutrients to support brain development and function [54]. Several studies support the potential benefits of specific dietary interventions, such as implementing a gluten-free lactose-free (GFCF) diet, which may help improve behavioral and digestive symptoms in some children with ASD. In addition, supplementation with omega-3 fatty acids, vitamins, and minerals (e.g., magnesium and zinc) have been proposed as potentially beneficial strategies to support neurologic health and alleviate ASD-related symptoms [55]. However, the effectiveness of dietary and nutritional interventions may vary by individual and more scientific research is needed to gain a deeper understanding of their long-term effects on children with ASD. Before implementing any dietary intervention, it is recommended to consult with a physician or nutritional expert to ensure that the individual needs of the child are met and to avoid malnutrition. In combination, dietary and nutritional interventions can be used as part of a comprehensive treatment plan for ASD, complementing traditional behavioral and educational interventions.

## Social and educational integration

### Educational integration of children with ASD

Educational integration of children with ASD is an inclusive educational practice that seeks to integrate children with ASD into the mainstream educational system to learn and grow with their typically developing peers. This integration model emphasizes individualized learning plans and adaptive teaching strategies to meet the unique needs of children with ASD while promoting their social inclusion and emotional development. Through educational integration, children with ASD are provided with opportunities to interact with other children, which is essential for them to learn social skills, enhance their communication abilities, and improve their ability to adapt to society. To support the successful integration of children with ASD, schools often provide special education services such as speech and language therapy, occupational therapy, and behavioral interventions, which take place in classroom settings to ensure their academic and social progress. Educational inclusion is not only beneficial for children with ASD, but it also helps to foster a sense of inclusion and diversity among their peers. By learning and playing together, all children learn to respect and understand differences, laying the foundation for a more inclusive society. However, effective integrated education requires close collaboration among teachers, parents and professionals, as well as the availability of appropriate resources and support systems [56].

### Social integration and employment of adults with ASD

The social integration and employment of adults with ASD is a current focus of attention in ASD research and social services. For many adults with ASD, social integration challenges include establishing stable relationships, participating in community activities, and finding and keeping a job. Although adults with ASD may have unique skills and interests in specific areas, social communication deficits and fixed patterns of behavior may make it difficult for them in traditional work settings. In recent years, more and more organizations and businesses have begun to recognize the value of diversity and inclusion and are working to create work environments that are better suited for adults with ASD. This includes providing flexible work arrangements, clear communication guidelines, and individualized support measures such as workplace co-worker support and professional career counseling. In addition, social service programs and non-profit organizations offer training and job readiness programs specifically designed for adults with ASD to help them develop necessary vocational skills and social competencies. Through these efforts, adults with ASD will not only be able to find jobs that meet their interests and abilities, but also find a place for themselves in society, enhancing their independence and life satisfaction. However, the realization of this goal requires sustained social awareness-raising and the construction of an ASD-friendly environment [57].

## Future research directions

### Application of precision medicine in ASD treatment

The application of precision medicine in the treatment of ASD represents a paradigm of a personalized treatment strategy that aims to tailor the treatment plan to each patient's genetic information, biomarkers, history of environmental exposure, and lifestyle factors. The philosophy behind this approach is that, although ASD is classified as a spectrum, each patient's etiology, symptoms, and their severity are different, and therefore treatment should be highly individualized [58, 59]. By fully sequencing a patient's genome, scientists and physicians can identify specific genetic variants that may affect ASD symptoms, allowing them to develop targeted treatments. For example, if a particular ASD patient's symptoms are linked to an abnormality in a specific metabolic pathway, that pathway could be modulated through dietary adjustments, nutritional supplements, or specific medications with a view to improving symptoms. In addition, precision medicine involves the consideration of environmental factors and personal behavior to ensure that treatment options are not only scientifically effective, but also appropriate to the patient's lifestyle. Although precision medicine is still in its early stages in the field of ASD, it offers great potential for delivering more personalized and effective treatment regimens, which are expected to significantly improve the quality of life of people with ASD [60].

### Prospects for emerging biotechnologies

Emerging biotechnologies in the field of ASD, such as gene editing, stem cell therapies, and biomarker development, are opening up new possibilities for treating and understanding ASD. Gene editing technologies, particularly the CRISPR-Cas9 system, provide researchers with the means to precisely modify genetic variants associated with ASD, promising to reveal how specific genetic variants affect brain development and function, thereby providing clues for the development of targeted therapies [61]. Stem cell therapies utilize a patient's own induced pluripotent stem cells (iPSCs) to study the pathomechanisms of ASD by mimicking the neurodevelopmental process in vitro, as well as exploring potential cellular alternative treatments. In addition, the discovery of biomarkers facilitates early diagnosis and monitoring of disease progression, making personalized treatment possible [62]. In addition, induced pluripotent stem cell (iPSC)-derived brain organoids from ASD patients have emerged as a powerful tool for studying the neurodevelopmental abnormalities associated with ASD. These 3D, self-organizing models recapitulate key features of human brain development in vitro, allowing researchers to investigate the cellular and molecular mechanisms underlying ASD pathogenesis. By comparing brain organoids derived from ASD patients with those from healthy controls, researchers can identify alterations in neuronal differentiation, migration, and connectivity that may contribute to the development of ASD. Moreover, patient-derived brain organoids provide a personalized platform for drug screening and testing, enabling the identification of targeted therapies that can be tailored to an individual's genetic background. This approach has the potential to revolutionize the development of precision medicine strategies for ASD, by providing a more accurate and relevant model system for investigating disease mechanisms and testing novel therapeutic interventions. As the field continues to advance, iPSC-derived brain organoids are expected to play an increasingly important role in unraveling the complex etiology of ASD and guiding the development of personalized treatment strategies [63]. The development of these technologies has not only improved our understanding of the complex etiology of ASD, but also provided more precise and effective treatment options for ASD patients. Although most of these emerging biotechnologies are still in the research phase, they bring hope and anticipation for the future of ASD treatment and management. As research progresses and technology matures, it is expected that these innovative approaches will bring substantial benefits to individuals with ASD and their families.

### Interaction between social policy and ASD research

The interaction between social policy and ASD research is key to achieving better social inclusion and quality of life for individuals with ASD and their families. Effective social policies can provide the necessary financial support and legal framework for ASD research, promoting a deeper understanding of ASD and the development of new treatments. For example, policies can promote collaboration in interdisciplinary research, encourage the use of innovative technologies and methods, and support long-term follow-up studies. In addition, social policies play a crucial role in ensuring that ASD research results are translated into practical applications and that education, employment, and social services are provided to individuals with ASD. Through the development of inclusive education policies, employment assistance programs, and the provision of integrated social services, policies can help individuals with ASD realize their potential and better integrate into society. At the same time, advances in ASD research also provide a scientific basis for the development of more targeted and effective social policies, helping policymakers understand the needs of individuals with ASD and develop more precise support measures. Thus, there is a close interplay between social policy and ASD research, which together have contributed to the advancement of the understanding of ASD and coping strategies.

## Conclusion

### Limitations of the current research

Although significant progress has been made in ASD research, a number of key limitations remain. First, the etiology of ASD is extremely complex, involving genetic and environmental factors and their interactions, making it extremely challenging to identify specific etiologies and develop targeted treatment strategies. Second, the heterogeneity of ASD is reflected in the extreme variability of symptoms among patients, which makes it difficult to develop uniform diagnostic criteria and treatment approaches. In addition, most studies have focused on children, and adult patients with ASD have been relatively understudied, which limits the understanding of the full lifespan of ASD. In terms of research methodology, most current ASD research relies on small, short-term studies, which may affect the broad applicability of results and the assessment of long-term effectiveness. In addition, although advances in technology have provided new tools for ASD diagnosis and intervention, the popularization and application of these technologies still face economic and resource constraints. Finally, ASD research is unequal across the globe, with far more research conducted in resource-rich countries and regions than in resource-limited areas. This imbalance limits a comprehensive understanding of ASD in different cultural and social contexts. Therefore, to overcome these limitations, more interdisciplinary, cross-cultural, and long-term research, as well as global collaborations, are needed to deepen the understanding of ASD and improve the quality of life of individuals with ASD.

### Perspectives on future research

The outlook for future prevention and treatment of ASD points in a more individualized, integrated, and technology-driven direction. With a deeper understanding of the genetic and environmental factors of ASD, it is expected that more targeted interventions and therapeutic strategies will be developed that will be based on an individual's specific genetic background and pathologic characteristics. The application of precision medicine is expected to improve treatment outcomes, reduce unwanted side effects, and optimize resource allocation. Meanwhile, technological advances, particularly artificial intelligence, machine learning, and virtual reality, are expected to revolutionize the way ASDs are diagnosed, monitored, and treated. These technologies are capable of delivering customized learning and treatment programs that enhance the acceptability and effectiveness of interventions. In addition, interdisciplinary research will be strengthened, and social policies and public health strategies will focus more on early screening and intervention, as well as increasing public awareness and understanding of ASD. Most importantly, the future of ASD prevention and treatment will place greater emphasis on the needs of patients and families, promote social integration and employment of patients, and improve their quality of life. As society's awareness of diversity and inclusion increases, individuals with ASD will receive more support and respect and enjoy fuller opportunities for social participation.

## Data Availability

No datasets were generated or analysed during the current study.
